# Public Health Lessons from Severe Acute Respiratory Syndrome a Decade Later

**DOI:** 10.3201/eid1906.121426

**Published:** 2013-06

**Authors:** Jeffrey P. Koplan, David Butler-Jones, Thomas Tsang, Wang Yu

**Affiliations:** Emory University, Atlanta, Georgia, USA (J.P. Koplan);; Public Health Agency of Canada, Ottawa, Ontario, Canada (D. Butler-Jones);; Centre for Health Protection, Hong Kong, People’s Republic of China (T. Tsang);; Chinese Center for Disease Control and Prevention, Beijing, China (W. Yu)

**Keywords:** severe acute respiratory syndrome, SARS, coronavirus, viruses, disease threats, public health, national public health institutes

## Abstract

The outbreak of severe acute respiratory syndrome in 2002–2003 exacted considerable human and economic costs from countries involved. It also exposed major weaknesses in several of these countries in coping with an outbreak of a newly emerged infectious disease. In the 10 years since the outbreak, in addition to the increase in knowledge of the biology and epidemiology of this disease, a major lesson learned is the value of having a national public health institute that is prepared to control disease outbreaks and designed to coordinate a national response and assist localities in their responses.

After an index case and ongoing transmission in Guangzhou, People’s Republic of China, in late fall 2002, the world experienced a widespread multicountry and multifocal outbreak of a new, virulent, transmissible respiratory illness in 2003. The causal agent of this disease was found to be a coronavirus, and the disease was named severe acute respiratory syndrome (SARS) (*1*). We are approaching the tenth anniversary of this dramatic event, which challenged public trust and government and health system capacity to address public health issues. What have we learned and what lessons have we acted upon during these years?

The science of SARS (biologic, ecologic, and epidemiologic) has been considerably elucidated (*1*). The coronavirus has been structurally described, associated with likely animal reservoirs (bat and civet cat), and demonstrated to be transmitted by droplets and possibly by aerosolization and contact with contaminated fomites. Transmission seems relatively easy because the basic reproduction number (R_0_) is 2.2–3.7, but transmission is greatly enhanced by occasional supertransmitters. The number of deaths from SARS is considerable; the case-fatality rate is ≈10%. Masks and other personal protective gear, along with active surveillance, case detection, contact tracing, isolation, and quarantine, were effective measures in reducing transmission. Even a relatively limited outbreak cost cities and countries many billions of dollars in lost business and productivity in addition to the human and economic costs of the extraordinary health and public health measures that were needed to identify and contain the disease (*2*).

During and following the outbreak, 8 broader national and international policy, operational, and systems needs were identified by public health officials. The first need was stronger and more integrated coordination between animal and human public health. This need is currently being addressed in One Health efforts by the World Health Organization, the Food and Agriculture Organization, the World Organisation for Animal Health, the US Centers for Disease Control and Prevention (CDC), and the Public Health Agency of Canada (*3*). The second need was enhanced disease and symptom surveillance systems that would share information quickly within countries and across borders. The third need was capable and responsive public health laboratories whose crucial role in infectious disease outbreaks includes establishing the etiologic agent, confirming the diagnoses in clinically suspected cases, supporting surveillance activities, and providing insight into the most effective infection control practices. The fourth need was for infection control to be stressed constantly in all health care settings. The fifth need was for development of clear criteria for isolation and quarantine and evaluation of the effectiveness of such measures. Isolation and quarantine were applied inconsistently with varied and often vague criteria (travel from an infected country, fever, sitting in an airplane near a traveler suspected of having SARS). Several sites believed that their isolation and quarantine efforts were helpful in reducing disease spread (*4*,*5*). The sixth need was for making risk assessment and communication as necessary components of the public health skill set to be used continuously during a health threat to a population (*6*). The seventh need was a prompt and practiced public health response with broad geopolitical responsibility and authority, spanning and linking political jurisdictions from municipalities to states/provinces to national and global levels. This response is better performed when preparedness training and a national incident response system have been routinely incorporated into the public health system. The eighth need was for national public health institutes (NPHIs) that have value in preventing and controlling outbreaks and other health threats (*7*). Even in the absence of an NPHI, the success of such entities in time of health crisis argues for at least establishing a central focus for coordination and leadership with delegated responsibility and authority.

NPHIs are the linchpin of public health systems in >80 countries. These institutes are science-based organizations that lead and coordinate public health at the national level. In most instances, NPHIs are part of the government (usually under the Ministry of Health) or closely attached to it.

NPHIs vary greatly from multifunctional and multidisciplinary agencies such as the US CDC, the Robert Koch Institute (Berlin, Germany), the Chinese CDC (Beijing, China) the National Institute for Public Health and the Environment (RIVM) (Bilthoven, the Netherlands), Fundação Oswaldo Cruz (Rio de Janeiro, Brazil), and the Public Health Agency of Canada (Ottawa, Ontario, Canada) to more targeted ones such as the Health Protection Agency (London, UK) and the National Institute for Communicable Diseases (Johannesburg, South Africa). Yet, despite their differences in history, scope, and resources, NPHIs all provide core public health functions that improve the efforts of their countries to address health challenges within and beyond their borders. These functions include population health assessment, health protection (surveillance and response), disease and injury prevention, health promotion, and research (evidence to inform policies and programs). In many countries, the NPHI plays a major role in developing and supporting local capacity, at the municipal and state/provincial levels, through training, technical assistance, tools, guidelines, staff assignments, and financing.

Consolidating these functions—and the associated skills, disciplines, experience, and expertise—in an NPHI provides many benefits, 2 of which are particularly germane to acute public health threats. The first benefit is the ability to generate and share knowledge, data, and evidence to inform public health decisions and policies. The second benefit is increased capacity to mount a quick, decisive, and coordinated response during a public health emergency. More than 80 NPHIs are organizationally linked through the International Association of National Public Health Institutes (IANPHI) (*8*), which promotes creation of new NPHIs (in Mozambique, Guinea-Bissau, and El Salvador) and assists in expanding the breadth and depth of existing NPHIs (e.g., in Uganda, Nigeria, Togo, Morocco, Ghana, Côte d’Ivoire, Rwanda, Bangladesh, Ethiopia, Tanzania). The World Health Organization does not have a formal relationship with individual NPHIs but has developed a partnership with IANPHI, including frequent communication and joint programmatic activities.

SARS provided an opportunity to recognize the value of the effectiveness of NPHIs when present and the risk for added toll of illness when absent. Thus, several countries involved in the outbreak subsequently saw fit to establish an NPHI (Hong Kong and Canada) or greatly strengthen an existing one (China). Such institutes are a positive legacy of the 2003 outbreak.

Likewise, in the past 10 years, NPHIs have played a major role in the response to many other public health crises, varying from natural disasters to disease outbreaks to addressing chronic noncommunicable diseases (e.g., controlling a widespread *Escherichia coli* outbreak; Robert Koch Institute); evaluating the effects of radiation exposure (NPHI, Tokyo, Japan); identifying and controlling measles outbreaks (Institut de Veille Sanitaire, Saint-Maurice, France); detecting Ebola virus (Uganda Virus Research Institute, Entebbe, Uganda, and National Institute for Communicable Diseases, South Africa); addressing a new influenza (H1N1) subtype outbreak (Instituto Nacional de Salud Publica, Mexico City, Mexico, in collaboration with the Public Health Agency of Canada and the US CDC), solving a milk contamination puzzle and promoting anti-tobacco efforts (Chinese CDC); dealing with health issues related to flooding (National Institute of Health, Bangkok, Thailand), and describing the epidemiology of Nipah virus (Institute of Epidemiology, Disease Control and Research, Dhaka, Bangladesh).

These examples demonstrate the value added by NPHIs worldwide. In addition to their major national role, by grouping organizationally through IANPHI, NPHIs also have a bond for coordination of efforts and mutual assistance that promotes their overall performance and technical development. When emerging infectious diseases, such as SARS, which become local, national, or global threats, are considered, the value and need for developing and strengthening NPHIs could be one of the major lessons learned and applied from the SARS experience of 10 years ago.
